# The independent contribution of miRNAs to the missing heritability in CYP3A4/5 functionality and the metabolism of atorvastatin

**DOI:** 10.1038/srep26544

**Published:** 2016-05-23

**Authors:** Ju-E Liu, Bin Ren, Lan Tang, Qian-Jie Tang, Xiao-Ying Liu, Xin Li, Xue Bai, Wan-Ping Zhong, Jin-Xiu Meng, Hao-Ming Lin, Hong Wu, Ji-Yan Chen, Shi-Long Zhong

**Affiliations:** 1 Department of Pharmacy, The First Affiliated Hospital, Sun Yat-sen University, Guangzhou, Guangdong 510080, China; 2 Medical Research Center, Guangdong General Hospital, Guangzhou, Guangdong 510080, China; 3 Department of Pharmaceutics, School of Pharmaceutical Sciences, Southern Medical University, Guangzhou 510515, China; 4 Institute of Chinese medical science, Guangdong TCM key Laboratory for metabolism, Guangdong Pharmaceutical University, Guangzhou 510006, China; 5 Department of Pharmacology, School of Pharmaceutical Sciences, Guangzhou Medical University, Guangzhou 511436, China; 6 School of Pharmaceutical Science, Sun Yat-Sen University, Guangzhou, Guangdong 510006, China; 7 Department of Hepatobiliary Surgery, Sun Yat-Sen Memorial Hospital, Sun Yat-sen University, Guangzhou 510120, China; 8 Guangdong Cardiovascular Institute, Guangdong Academy of Medical Sciences, Guangzhou, Guangdong 510080, China

## Abstract

To evaluate the independent contribution of miRNAs to the missing heritability in CYP3A4/5 functionality and atorvastatin metabolism, the relationships among three levels of factors, namely (1) clinical characteristics, CYP3A4/5 genotypes, and miRNAs, (2) CYP3A4 and CYP3A5 mRNAs, and (3) CYP3A activity, as well as their individual impacts on atorvastatin metabolism, were assessed in 55 human liver tissues. MiR-27b, miR-206, and CYP3A4 mRNA respectively accounted for 20.0%, 5.8%, and 9.5% of the interindividual variations in CYP3A activity. MiR-142 was an independent contributor to the expressions of CYP3A4 mRNA (partial R^2^ = 0.12, P = 0.002) and CYP3A5 mRNA (partial R^2^ = 0.09, P = 0.005) but not CYP3A activity or atorvastatin metabolism. CYP3A activity was a unique independent predictor of variability of atorvastatin metabolism, explaining the majority of the variance in reduction of atorvastatin (60.0%) and formation of ortho-hydroxy atorvastatin (78.8%) and para-hydroxy atorvastatin (83.9%). MiR-27b and miR-206 were found to repress CYP3A4 gene expression and CYP3A activity by directly binding to CYP3A4 3′-UTR, while miR-142 was found to indirectly repress CYP3A activity. Our study indicates that miRNAs play significant roles in bridging the gap between epigenetic effects and missing heritability in CYP3A functionality.

Atorvastatin (AT) is the first-line lipid-lowering drug for the majority of people with dyslipidemia and one of the most widely prescribed drugs worldwide. However, AT shows great interindividual variation, such as 6-fold variability in maximum concentration among subjects receiving 80 mg of AT^1^.

AT is mainly metabolized by cytochrome P450 (CYP) 3A^2^, and ortho-hydroxy atorvastatin (OAT) and para-hydroxy atorvastatin (PAT) are two metabolites of AT in microsome incubation systems *in vitro*^2^,^3^,^4^. CYP3A4 and CYP3A5 are the most prominent of the CYP3A enzymes and have been reported to contribute to biotransformation of AT *in vitro*^2^,^4^,^5^. Genetic polymorphisms of CYP3A5 may affect the lipid-lowering responses and pharmacokinetic profiles of statins^6^,^7^. Previous studies have reported the importance of the CYP3A subfamily in the metabolism of statins^4^,^6^,^8^,^9^. Other phase I metabolic enzymes, such as CYP2C8, CYP2C9 and CYP2C19, and phase II metabolic enzymes, such as UGT1A1 and UGT1A3, are also involved^10^,^11^,^12^. However, no consistent association between the genetic variation of CYP3A4/5 and pharmacokinetic/pharmacodynamic responses to AT has been found to date.

Missing heritability is evident in CYP3A functionality. CYP3A4 and CYP3A5 are typically regulated by a multitude of intrinsic and extrinsic factors, resulting in significant interindividual variability of expression and function with clinical relevance that can not be explained solely by genetic variations^13^,^14^. Epigenetic mechanisms to regulate metabolic enzyme genes may provide new insights into the causes of missing heritability of CYP3A on AT metabolism^15^. In particular, differentially expressed microRNA (miRNA) may be an important cause of variation in enzyme functionality^16^.

MiRNAs have been shown to play important roles in cell cycle, numerous disease states, including cancer and angiocardiopathy, and drug metabolism^17^. Some miRNAs directly or indirectly regulate the expression of xenobiotic metabolizing cytochrome P450 enzymes, the ATP-binding cassette, and solute carrier transporters or nuclear receptors^18^. MiRNA expression was recently reported to contribute to the regulation of the expression of drug transporters and nuclear receptors linked to P450 enzymes. Yuki^19^ discovered that decreased expression of miR-27b caused high expression of CYP1B1 protein in cancerous tissues. Yu^20^ found that CYP3A4 gene expression might be regulated by miR-27b at both the transcriptional and posttranscriptional levels.

In the present study, we reveal a relationship between miRNAs and CYP3A4/5 gene expression and activity, which bridges the missing heritability of CYP3A4/5 activity. Furthermore, the independent contributions of CYP3A4/5 genotype, gene expression, and activity, as well as miRNAs, to AT metabolism in human liver tissues are investigated.

## Results

### Patient characteristics of liver samples

Baseline clinical characteristics and their impact on CYP3A4/5 gene expression and CYP3A activity in 55 patients are shown in Supplementary Table S1. The donors of liver tissues were aged 20 to 77 years with a median age of 48 years. No significant correlation was found between the clinical characteristics of patients and CYP3A4/5 gene expression and activity in liver tissues (all P > 0.05). Being male was associated with decreased reduction in AT in the liver microsome metabolic system (B = −10.49, P = 0.017). No significant correlation was found between other clinical characteristics of patients and AT metabolism in liver microsomes (all P > 0.05).

### Relationships among genotypes, CYP3A4/5 gene expression, and CYP3A activity in human liver microsomes (HLM)

**Figure 1:**
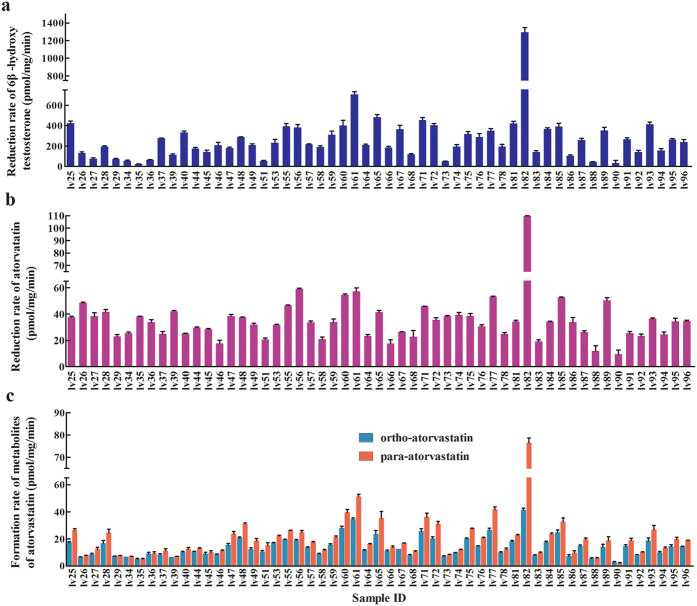
Metabolism of testosterone and atorvastatin in human liver microsomes. (**a**) Formation rate of 6β-hydroxy testosterone; (**b**) Reduction rate of atorvastatin; (**c**) Formation rate of ortho- and para-hydroxy atorvastatin. Data shown are the average mean ± S.D. of three independent replicates.

Substantial interindividual differences in CYP3A4/5 expression and CYP3A activity were observed. The CYP3A activity in liver tissues varied widely with a 60-fold range of formation rate of testosterone 6β-hydroxylation from 23 pmol/mg/min to 1,293 pmol/mg/min (Fig. 1a), consistent with published data^21^.

**Figure 2:**
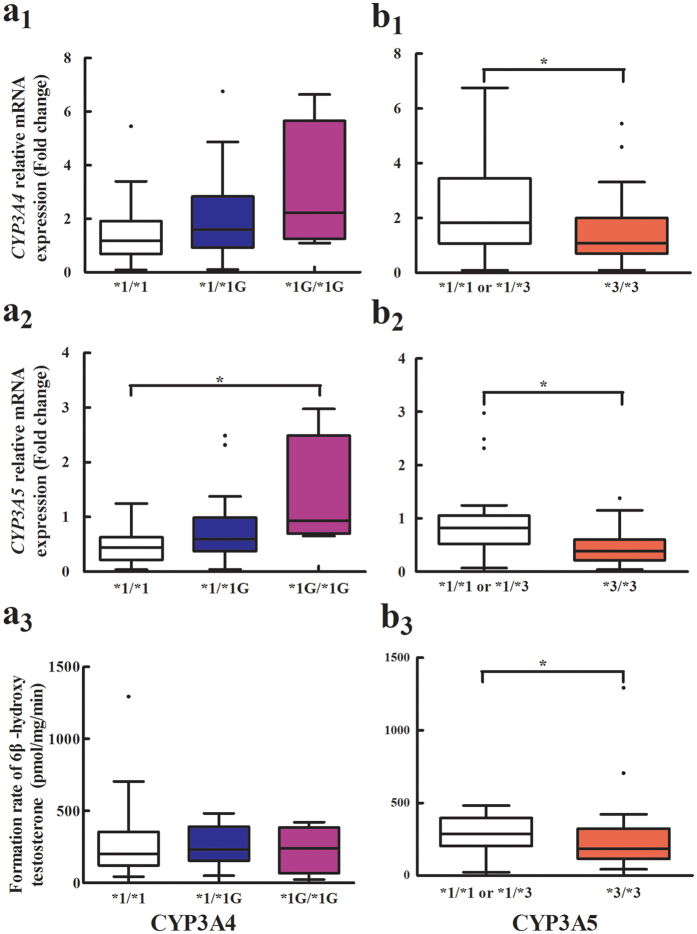
Effects of the CYP3A4*1G (G > A) and CYP3A5*3 (A > G) single-nucleotide polymorphism (SNP) on CYP3A4/5 mRNA expression levels and CYP3A activity in human livers. The results are shown as box plot where the box represents the middle 50% of the data and the whiskers represents the spread of the remaining data. The line in the center represents the median. (**a**_**1**_) Relative CYP3A4 mRNA levels were plotted against the CYP3A4*1G (G > A) genotype; (**a**_**2**_) Relative CYP3A5 mRNA levels were plotted against the CYP3A4*1G (G > A) genotype; (**a**_**3**_) CYP3A activity were plotted against the CYP3A4*1G (G > A) genotype; (**b**_**1**_) Relative CYP3A4 mRNA levels were plotted against the CYP3A5*3 (A > G) genotype. (**b**_**2**_) Relative CYP3A5 mRNA levels were plotted against the CYP3A5*3 (A > G) genotype; (**b**_**3**_) Relative CYP3A activity were plotted against the CYP3A5*3 (A > G) genotype. *P < 0.0.5.

CYP3A4*1G was associated with increased CYP3A5 mRNA expression (0.48 ± 0.35 for *1/*1, 0.76 ± 0.62 for *1/*1G, 1.37 ± 1.08 for *1G/*1G; P = 0.033) (Fig. 2a2). No significant difference was observed between CYP3A4*1G polymorphism and CYP3A4 mRNA expression and activity (P > 0.05) (Supplementary Table S1, Fig. 2a1,a3). CYP3A4*1B and CYP3A4*22 alleles were not observed in any of the 55 liver samples. CYP3A4 and CYP3A5 mRNA were significantly higher in subjects with at least one CYP3A5*1 allele than in those with the *3/*3 genotype (P = 0.044, 0.001, respectively) (Supplementary Table S1, Fig. 2b1,b2). CYP3A activity in liver samples carrying at least one *1 allele was significantly higher than that in samples with the *3/*3 genotype (285.9 ± 124.9 vs. 243.6 ± 236.6, P = 0.029) (Fig. 2b3).

**Figure 3:**
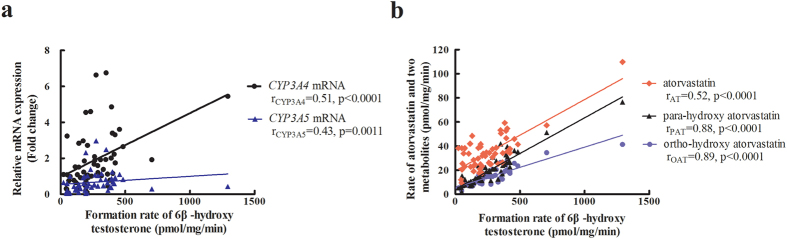
Correlation between CYP3A enzyme activity and CYP3A4/5 gene expression in 55 individual liver microsomes. Spearman correlation analysis was performed to analyze the association of CYP3A enzyme activity with CYP3A gene expression and atorvastatin metabolites. (**a**) CYP3A enzyme activity was significantly associated with CYP3A4/5 gene expression. (**b**) CYP3A enzyme activity were significantly associated with atorvastatin metabolism. r_CYP3A4_, Spearman correlation (r value) of CYP3A enzyme activity and CYP3A4 mRNA. r_CYP3A5_, Spearman correlation of CYP3A enzyme activity and CYP3A5 mRNA. r_AT_, Spearman correlation of CYP3A enzyme activity and reduction rate of atorvastatin. r_OAT_, Spearman correlation of CYP3A enzyme activity and formation rate of ortho-hydroxy atorvastatin. r_PAT_, Spearman correlation of CYP3A enzyme activity and formation rate of para-hydroxy atorvastatin. N = 55.

CYP3A activity was positively associated with CYP3A4 mRNA (Spearman r = 0.51, P = 0.0002, 95%CI: 0.28–0.69) and CYP3A5 mRNA (Spearman r = 0.43, P = 0.0015, 95%CI: 0.18–0.63) (Fig. 3a).

### Relationships among miRNAs and CYP3A4 mRNA, CYP3A5 mRNA, and CYP3A activity in liver tissues

Among the 13 miRNAs tested, miR-27b, miR-130a, miR-21, and miR-142 were significantly negatively associated with CYP3A4 mRNA in the human liver. MiR-142, miR-130a, and miR-21 were marginally negatively associated with CYP3A5 mRNAs. MiR-27b, miR-130a, and miR-27a were also negatively correlated with CYP3A activity in human liver tissues. MiR-142, miR-21 (r = −0.31, P = 0.020, FDR = 0.043) were negatively associated with CYP3A activity. MiR-206 was not associated with CYP3A4 mRNA or CYP3A5 mRNA but was negatively correlated with CYP3A activity (Supplementary Table S2).

### Impact of CYP3A4/5 genotypes, gene expression, and CYP3A activity on AT metabolism

Large variability in hydroxylation of AT was observed in the 55 liver samples study (Fig. 1b). The formation rates of OAT and PAT at 3.5 μM AT were 14.2 ± 7.5 and 19.6 ± 12.9 pmol/mg/min, respectively (Fig. 1c). The formation rates of OAT and PAT varied from 3 pmol/mg/min to 41 pmol/mg/minand from 2.1 pmol/mg/min to 76.6 pmol/mg/min, respectively. The high interindividual variability of AT metabolism *in vitro* is consistent with previous findings on the *in vivo* pharmacokinetic studies of AT^1^.

**Figure 4:**
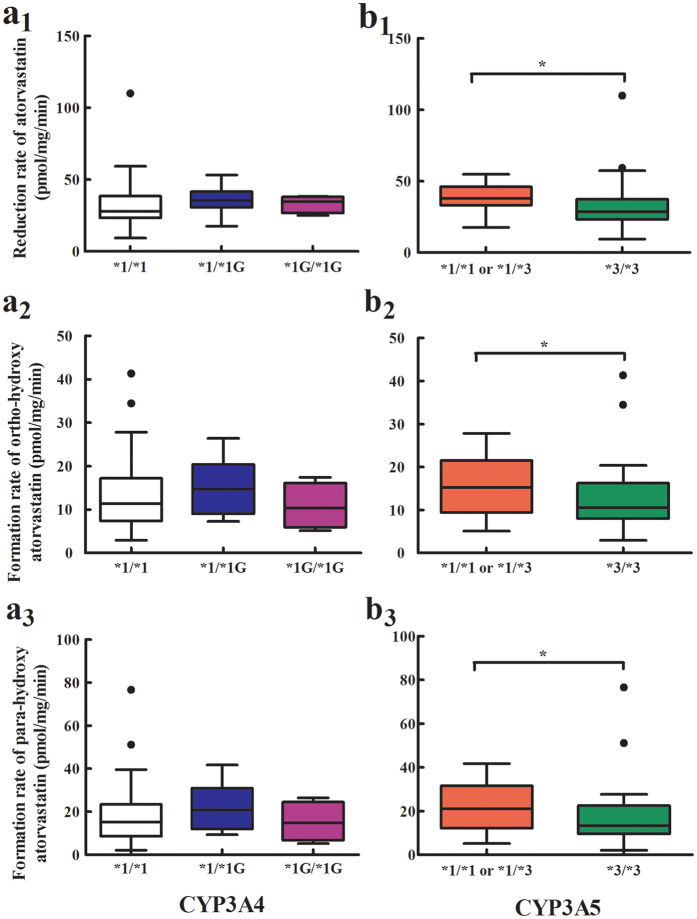
Association of the CYP3A4*1G and CYP3A5*3 single-nucleotide polymorphism with atorvastatin and its metabolites. (**a**_**1**_**,b**_**1**_) Reduction rate of **atorvastatin**; (**a**_**2**_**,b**_**2**_) Formation rate of ortho-hydroxy atorvastatin; (**a**_**3**_**,b**_**3**_) Formation rate of para-hydroxy atorvastatin.

No significant effect of CYP3A4*1G on AT metabolism was observed. CYP3A5*3 polymorphism was significantly associated with reductions in AT and the formation rates of OAT and PAT. The reduction rates of AT and formation rates of OAT and PAT were significantly lower in subjects with CYP3A5*3/*3 than in those with other genotypes (Fig. 4).

CYP3A4 gene expression was highly positively correlated with reductions in AT and the formation rates of OAT and PAT (r = 0.47, P = 0.0003; r = 0.46, P = 0.0004; and r = 0.44, P = 0.0007; Supplementary Fig. S1a_1_–a_3_). CYP3A5 gene expression was also highly positively correlated with reductions in AT and the formation rates of OAT and PAT (r = 0.33, P = 0.0137; r = 0.39, P = 0.0030; and r = 0.40, P = 0.0024; Supplementary Fig. S1b_1_–b_3_).

As expected, CYP3A enzyme activity exerted a strong impact on the reduction of AT and the formation rates of OAT and PAT in HLM. Higher CYP3A activity was associated with larger reductions in AT and the formation rates of OAT and PAT (r = 0.52, P < 0.0001; r = 0.89, P < 0.0001; and r = 0.88, P < 0.0001; Fig. 3b).

### Impact of miRNAs on atorvastatin metabolism

Among the 13 miRNAs studied, miR-27b, miR-206, and miR-21 were significantly negatively correlated with reductions in AT (r = −0.38, P = 0.004, FDR = 0.022; r = −0.42, P = 0.001, FDR = 0.013; r = −0.37, P = 0.005, FDR = 0.022, respectively). MiR-27b, miR-206, and miR-130a were significantly negatively correlated with the formation rates of OAT (r = −0.43, P = 0.001, FDR = 0.013; r = −0.36, P = 0.007, FDR= 0.030; r = −0.36, P = 0.007, FDR = 0.030, respectively). MiR-27b, miR-206, miR-21, miR-27a, and miR-130a were significantly negatively correlated with the formation rates of PAT (r = −0.46, P = 0.001; FDR= 0.013; r =−0.39, P = 0.003, FDR = 0.013; r = −0.35, P = 0.010, FDR= 0.033; r = −0.33, P = 0.014, FDR = 0.036; r = −0.39, P = 0.003, FDR = 0.013, respectively, Supplementary Table S2).

### Independent contribution of the genotype, miRNAs, gene expression, and activity of CYP3A4/5 to atorvastatin metabolism

Multivariate linear regression analysis showed that the presence of cancer, CYP3A4*1G polymorphism, and miR-142 were independent factors influencing the variability of CYP3A4 mRNA expression with the model R^2^ = 0.29. Liver cancer, CYP3A5*3 polymorphism, and miR-142 were independent factors for the variability of CYP3A5 mRNA with the model R^2^ = 0.35. CYP3A4 mRNA, miR-27b, and miR-206 were independent predictors of CYP3A activity, explaining 35.3% of the variance observed. CYP3A activity was the only independent predictor of the variability of AT metabolism, explaining the majority of the variance in reduction in AT (60.0%) and formation of OAT (78.8%) and PAT (83.9%) (Supplementary Table S3).

### Effects of overexpression or inhibition of five miRNAs on CYP3A4 and CYP3A5 mRNA

**Figure 5:**
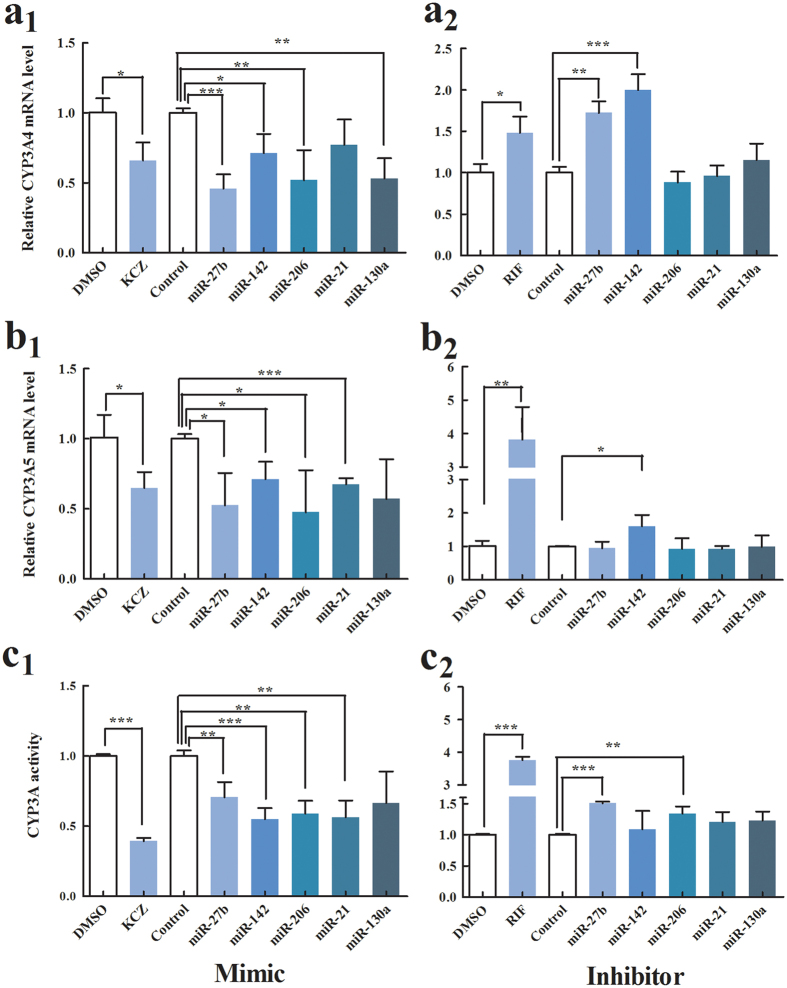
Effects of overexpression or inhibition of five miRNAs on CYP3A activity, and CYP3A4 and CYP3A5 gene expression. CYP3A activity after 48 h transfected with (**a**_**1**_) mimic or (**a**_**2**_) inhibitor of five miRNAs into human primary hepatocytes. CYP3A4 mRNA level 48 h after transfection with (**b**_**1**_) mimic or (**b**_**2**_) inhibitor of five miRNAs into HepG2 cells. CYP3A5 mRNA level 48 h after transfection with (**c**_**1**_) mimic or (**c**_**2**_) inhibitor for five miRNAs into HepG2 cell. *P < 0.05, **P < 0.001, ***P < 0.001.

To test whether CYP3A4 or CYP3A5 gene expression was regulated by miRNAs, we quantified the levels of CYP3A4 and CYP3A5 gene expression by qRT-PCR 48 h after transfection of 75 nM mimic or 100 nM miRNA inhibitor or control into HepG2 cells. Rifampin and ketoconazole were selected as induction and inhibition controls, respectively. As expected, we found that CYP3A4 and CYP3A5 gene expression was increased by rifampicin and decreased by ketoconazole. The relative level of CYP3A4 mRNA was significantly decreased by mimics of miR-27b, miR-142, miR-206 and miR-130a, compared with negative controls, while the level of CYP3A4 mRNA was increased by inhibitors of miR-27b and miR-142. The CYP3A5 mRNA level was significantly decreased by mimics of miR-27b, miR-142, miR-206 and miR-21, but increased by inhibitor of miR-142. These results suggest that miR-142 could independently inhibit CYP3A4 and CYP3A5 expression and that miR-27b could inhibit CYP3A4 expression (Fig. 5a,b).

### Effects of overexpression or inhibition of five miRNAs on CYP3A activity

To investigate the effects of miR-27b, miR-206, miR-21, miR-130a and miR-142 on regulation of CYP3A activity, 75 nM mimic or 100 nM inhibitor of miRNA or control was transfected into human primary hepatocytes using Lipofectamine 3000 (Invitrogen Life Technologies, USA). After 48 h, CYP3A activity was measured using the P450-Glo™ CYP3A4 assay (Promega, USA). We used rifampin and ketoconazole as induction and inhibition controls, respectively, and found that 25 μmol/l of rifampin significantly increased the expression of CYP3A4 activity, while 1 μmol/l of ketoconazole significantly decreased the expression of CYP3A4 activity compared with DMSO-treated cells. CYP3A enzyme activity was significantly decreased by transfection of mimics of miR-27b, miR-206, miR-21, and miR-142 into the human primary hepatocytes, while CYP3A enzyme activity was significantly increased by inhibitors of miR-27b and miR-206, compared to the control (Fig. 5c).

### Repressive regulation of CYP3A4 by miR-27b and miR-206

**Figure 6:**
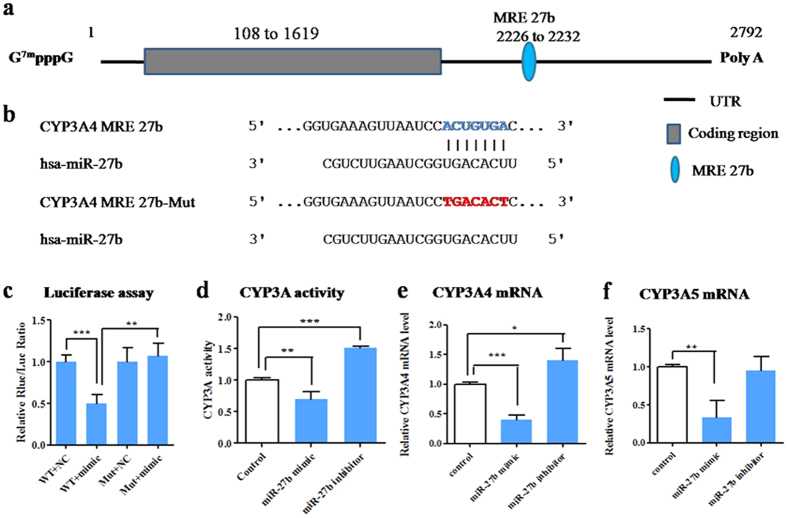
MiR-27b represses CYP3A activity by directly binding with CYP3A4 3′-UTR. (**a**) The predicted target sequence of miR-27b in human CYP3A4 mRNA. (**b**) The potential miR-27b recognition element with blue base (CYP3A4 MRE 27b) is located between bases 2226 and 2232. (**c**) The wild type or mutant reporter plasmids (100 ng) were co-transfected with miR-27b mimic or control into HEK293 cells. The *Renilla* luciferase activity for each construct was normalized with the firefly luciferase activity. (**d**) The effect of miR-27b mimic or inhibitor on CYP3A activity. (**e**) Relative CYP3A4 mRNA level. (**f**) Relative CYP3A5 mRNA level. The values of activity and mRNA level were measured 48 h after transfection with mimic or inhibitor for miR-27b. Each column represents the mean ± SD of three independent experiments. *P < 0.05, **P < 0.001, ***P < 0.001.

**Figure 7:**
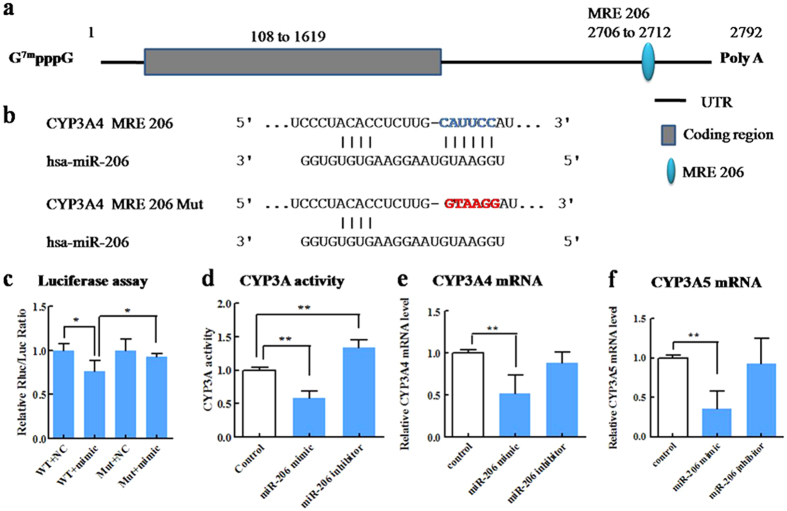
MiR-206 represses CYP3A activity by directly binding with CYP3A4 3′-UTR. (**a**) The predicted target sequence of miR-206 in human CYP3A4 mRNA. (**b**) The potential miR-206 recognition element with blue base (CYP3A4 MRE 206) is located between bases 2706 and 2712. (**c**) The wild type or mutant reporter plasmids (100 ng) were co-transfected with miR-206 mimic or control into HEK293 cells. The *Renilla* luciferase activity for each construct was normalized with firefly luciferase activity. (**d**) The effect of miR-206 mimic or inhibitor on CYP3A activity. (**e**) Relative CYP3A4 mRNA level. (**f**) Relative CYP3A5 mRNA level. The values of activity and mRNA level were measured 48 h after transfection with mimic or inhibitor for miR-206. Each column represents the mean ± S.D. of three independent experiments. *P < 0.05, **P < 0.001, ***P < 0.001.

To validate whether miR-27b, miR-206, miR-21, and miR-130a regulate CYP3A4 and miR-27b and miR-142 regulate CYP3A5 by directly targeting binding sites, wild-type and mutant versions of CYP3A4 3′-UTR and CYP3A5 3′-UTR were constructed and cloned downstream of a luciferase reporter gene. The luciferase reporter plasmid was co-transfected with mimics of the corresponding miRNA into HEK293 cells. Results showed that luciferase activity of the pRB/CYP3A4 wild-type plasmid was significantly decreased by co-transfection with mimic of miR-27b (54%, p < 0.01), whereas that of the pRB/CYP3A4 MRE-27b-Mut plasmid, which conained a mutant recognition site, was not affected (Fig. 6). These results indicate that miR-27b functionally recognizes CYP3A4 MRE27b to decrease CYP3A4 expression. Dual-luciferase reporter assay showed that miR-206 mimic transfection significantly (p < 0.05) decreased the luciferase activity of the group transfected with the pRB/CYP3A4 wild-type plasmid but had no effect on the group transfected with pRB/CYP3A4 MRE-206-Mut (Fig. 7). These results indicate that miR-206 can functionally target CYP3A4 MRE206 to decrease CYP3A4 expression. However, the luciferase activities of neither the pRB/CYP3A4 wild-type nor the mutant plasmid were affected by miR-21 and miR-130a (Supplementary Fig. S2a,b), indicating that miR-21 and miR-130a did not target the predicted binding sites in the CYP3A4 3′-UTR. Similarly, the luciferase activities of both the pRB/CYP3A5 wild-type and mutant plasmids were unaffected by miR-27b and miR-142 (Supplementary Fig. S2c,d). These results show that miR-27b and miR-142 do not target the predicted CYP3A5 MRE.

**Figure 8:**
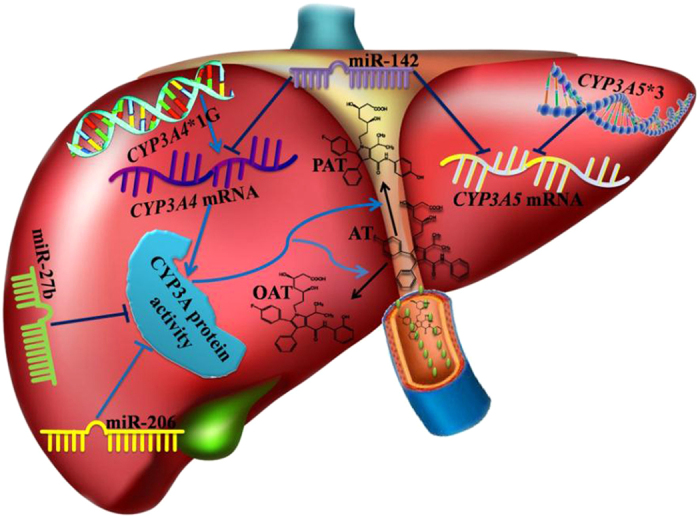
Summary of the independent contributions of genotype, miRNAs, gene expression, and activity of CYP3A enzyme to atorvastatin metabolism.

The independent contributions of genotype, miRNAs, gene expression, and activity of CYP3A4/5 to AT metabolism based on the above relationships is summarized in Fig. 8.

## Discussion

In this study, we investigated the independent contributions of three levels of factors to AT metabolism using direct and indirect approaches and across the central dogma of biology. The first level includes clinical characteristics, CYP3A4/5 genotype, and miRNAs targeting the CYP3A4/5 mRNA sequence, the second level includes CYP3A4 mRNA and CYP3A5 mRNA, and the third level includes CYP3A activity. Among the three levels of factors studies, CYP3A activity was the only independent predictor of variability of AT metabolism, explaining most of the variance in reductions in AT (60.0%) and formation of OAT (78.8%) and PAT (83.9%). Interindividual differences in CYP3A activity were affected not only by CYP3A4 gene expression but also by miR-27b and miR-206. Our study indicates that miRNAs play a significant role in bridging the gap between epigenetic effects and missing heritability in CYP3A functionality, which further supports the notion that CYP3A activity is an important factor in AT metabolism.

Considering the large interindividual variability and missing heritability of CYP3A activity, identification of the factors influencing CYP3A activity can be useful to predict clinical drug responses. Our study demonstrated that 35.5% of the interindividual variation in CYP3A activity maybe predicted by CYP3A4 mRNA levels, which are regulated by miR-27b, and miR-206. Thus, CYP3A4 is the predominant form of Cytochrome P contributing to most of the hepatic CYP3A activity in humans, consistent with published data. An *in vitro* study reported that CYP3A4 and CYP3A5 were responsible for 85% and 15%, respectively, of AT metabolism^4^.

Although CYP3A5 plays a limited role in statin metabolism, results of the Mann-Whitney U test showed that CYP3A5*3 polymorphism was significantly associated with CYP3A4 and CYP3A5 gene expressions, CYP3A activity, and AT metabolism. This finding is in agreement with previous observations^22^,^23^. CYP3A5*3 polymorphism has been reported to contribute to interindividual differences in the clearance^24^ and response to some statins, including AT^6^. Patients who develop myalgia while taking AT are more likely to experience a greater degree of muscle damage if they express two copies of CYP3A5*3^25^.

CYP3A5*3 was independently associated with CYP3A5 gene expression, but not CYP3A4 gene expression, CYP3A activity, or AT metabolism, which suggests that CYP3A5*3 has indirect effect on AT activity through gene expression and indirect impact on AT metabolism. The CYP3A5*3 allele causes alternative splicing and protein truncation and results in the absence of CYP3A5 and loss of CYP3A5 activity^22^.

Our study demonstrated that miRNAs play an important role in CYP3A gene expression and CYP3A activity. In this study, we investigated 12 miRNAs predicted to target CYP3A4/5 mRNA and a negative control miRNA (miR-1260b). The expression levels of four related miRNAs, namely, miR-27b, miR-21, miR-130a, and miR-142, were significantly negatively associated with CYP3A4 mRNA expression level. Three miRNAs predicted to target the CYP3A5 sequence, namely, miR-21, miR-130a, and miR-142, were marginally negatively associated with CYP3A5 mRNA level in 55 human liver tissues. However, only miR-142 was identified to be an independent contributor to variability of CYP3A4 and CYP3A5 mRNA expression. MiR-142 was associated with CYP3A activity but was not an independent factor. Data from the *in vitro* model showed that miR-142 could inhibit CYP3A4 and CYP3A5 gene expression as well as CYP3A activity. Nevertheless, the luciferase reporter assay revealed that CYP3A5 is not directly regulated by miR-142. Together, these data indicate that the effect of miR-142 on CYP3A activity occurs through inhibition of CYP3A4 and CYP3A5 transcription, and that the inhibition does not occur by directly targeting CYP3A4 and CYP3A5 mRNA, but rather by an indirect pathway.

Multiple linear regression with clinical characteristics, genotypes, and miRNAs showed that only miR-27b was independently associated with the formation of OAT and PAT, and marginally associated with AT reduction. However, when considering CYP3A4/5 mRNA expression and activity as parameters, no parameters of clinical characteristics, genotypes, and miRNAs were independently associated with reductions in AT and formation of OAT and PAT. MiR-27b and miR-206 were also identified to be independent contributors to variability of CYP3A activity in liver tissues.

To validate the above results and elucidate potential mechanisms, the effects of overexpression and inhibition of these miRNAs were evaluated in luciferase reporter assays. Results indicated that miR-27b could repress CYP3A4 gene expression and CYP3A activity through direct binding to the CYP3A4 3′-UTR. Our results are consistent with previous reports by Pan, YZ *et al*.^20^. MiR-206 could repress CYP3A4 gene expression by directly binding with the CYP3A4 3′-UTR, and there by decrease CYP3A activity. Although there was no miR-206 binding site in the CYP3A5 mRNA sequence, miR-206 could also repress CYP3A5 gene expression. The mechanism underlying this phenomenon needs to be clarified in a future study.

Preview studies revealed that miRNAs play an important role in the transcription and translation of CYP3A. Vuppalanchi R *et al*.^26^ analyzed the association between hepatic CYP3A activity and miRNA microarray expression profiles in cirrhotic and normal liver tissue, and found that miR-155 negatively correlated with hepatic CYP3A activity. Wei Z *et al*.^27^ found that hsa-miR-577, hsa-miR-1, hsa-miR-532-3p and hsa-miR-627 could significantly down-regulate the translation efficiency of CYP3A4 mRNA in the liver. In our study, we investigated the effects of miRNA not merely on CYP3A4 but also on CYP3A5. Our results indicated that CYP3A activity could be repressed by miR-27b and miR-206 via translational regulation and by miR-142 through transcriptional inhibition of CYP3A4 and CYP3A5.

Our study is different from previous studies in these aspects: (1) Candidate miRNA selection— all miRNAs were predicted to target CYP3A4 or CYP3A5 mRNA sequence and previously experimentally verified to target enzyme genes involved in atorvastatin metabolism (e.g., CYP3A4, CYP3A5, CYP2C8, UGT1A1 and UGT1A3), factors regulating CYP3A4 function (e.g., nuclear receptors), and factors involving inflammatory signaling; (2) We investigated the independent contribution of miRNAs, as well as CYP3A4/5 genotypes, to CYP3A4 or CYP3A5 mRNA and CYP3A activity and showed that miRNAs play a significant role in bridging the gap between epigenetic effects of miRNA and missing heritability in CYP3A functionality; (3) The contribution of miRNA to the missing heritability in CYP3A was investigated to elucidate the effect of miRNAs on atorvastatin metabolism.

In conclusion, this study showed that genetic and epigenetic factors could contribute to interindividual differences in CYP3A activity and that CYP3A activity is the main contributor to variation in AT metabolism *in vitro*.

## Materials and Methods

### Ethics statement

All experimental protocols are in accordance with the Declaration of Helsinki and its subsequent revisions and was approved by the Medical Ethical Review Committee of Sun Yat-Sen Memorial Hospital (Guangzhou, China) and Guangdong General Hospital (Guangzhou, China).

### Liver tissues and microsome preparation

Liver tissues from distant non-cancerous liver tissues were obtained surgically or from liver resection from individuals with benign liver diseases at Sun Yat-Sen Memorial Hospital (Guangzhou, Guangdong, China) after informed consent was received between September 2012 and May 2015.

Specimens for microsome extraction were rapidly prepared using GENMED A Solution (GENMED Scientific Inc., USA) and stored in liquid nitrogen. Specimens for mRNA measurement were stabilized and stored at −80 °C in RNAlater^®^ solution (Qiagen, Germany).

The procedure for human liver microsome preparation was modified from a procedure published previously^28^,^29^. Briefly, frozen liver tissues were weighed and thawed immediately at 37 °C for less than 1 min. Liver samples were homogenized using amotor-operated homogenizer in homogenization buffer in a special glass. The homogenized samples were then centrifuged at 10,400 rpm for 15 min at 4 °C to remove cell debris, lysosomes, and mitochondria. The precipitate was discarded, and the supernatant was subjected to further centrifugation at 35,000 rpm for 60 min at 4 °C. The microsomal pellet was obtained and dissolved by adding 0.25 M sucrose, subsequently subpackaged into microtubes, and stored at −80 °C. Protein concentrations were determined using the Bradford Protein Assay Kit (Beyotime, China).

### CYP3A enzyme activity

CYP3A activity was assessed by using testosterone as a probe, and the formation rate of 6β-hydroxylation testosterone (6β-OHT) was quantified from HLMs. In brief, testosterone (20 μM) was incubated with HLM for 20 minutes. The incubation procedures for the CYP reaction were the same as those published previously^30^. All incubations were performed in triplicate. The samples were analyzed using ultra-performance liquid chromatography with ultraviolet detection (Supplementary Fig. S3). The CYP3A enzyme activity of microsomes was determined based on the formation rate of 6β-OHT. Determinations of the optimum incubation conditions, incubation time, and concentration of microsome proteins were performed (Supplementary Fig. S4).

### Atorvastatin metabolism in HLM

The incubation conditions were modified from Fujino *et al*.^31^. Briefly, the incubation mixture with AT (3.5 μM) was incubated for 60 minutes. A high-performance liquid chromatography–tandem mass spectrometry assay was developed and validated for simultaneous determination of AT, its active metabolites, and internal standard of carbamazepine in microsomal incubation mixtures. Briefly, chromatography was performed using a Synergi 4u Fusion-RP 80A (150 mm × 2.0 mmi.d., Phenomenex, USA) at 40 °C. The mobile phase consisting of acetonitrile with 0.1% formic acidand water (60:40, v/v) was delivered at a flow rate of 0.25 mL/min. Mass spectrometric detection was performed on an API 4000 triple quadrupole instrument (Applied Biosystems, Foster City, CA, USA) in positive electrospray ionization mode. The precursor-to-product ion reactions monitored had m/z ratios ranging from 559.4 to 440.2, 575.4 to 440.1, and 237.2 to 194.2 for AT, OAT and PAT, and carbamazepine, respectively (Supplementary Figs S5 and S6).

To determine the apparent K_m_ and V_max_ of metabolite formation, pooled liver microsomes were incubated with1, 2, 8, 15, 30, 45, 60, 90, 120, or 150 μM AT (n = 3 for each concentration). Enzyme kinetics and the effect of AT incubation time and microsomal protein concentration on AT metabolism by human liver microsomes are shown in Supplementary Fig. S7.

### Genotyping for CYP3A4*1G, *B, *22, and CYP3A5*3

Genomic DNA was isolated from liver tissue using the TIANamp Genomic DNA Kit (Tiangen Biotech Co., Ltd, Beijing, China). The quality and quantity of the DNA was assessed using a NanoDrop 2000 Spectrophotometer (Thermo Scientific, USA).

All liver samples were genotyped for the CYP3A5*3 (6986A>G) allele by allelic discrimination with a TaqMan SNP assay using the ABI Vii7 Real-Time PCR system (Applied Biosystems, USA). The primers used were 5′-AACATTATGGAGAGTGGCATAGGAG-3′ (forward) and 5′-TGTAATCCATACCCCTAGTTGTACGA-3′ (reverse). The TaqMan MGB probes were FAM-TGTCTTTCAATATCTCTTC-MGB and HEX-TTTTGTCTTTCAGTATCTC-MGB. The following thermal profile was used for PCR: initial denaturation at 95 °C for 10 min, followed by 40 cycles of denaturation at 95 °C for 15 s, and extension at 60 °C for 1 min. Genotyping accuracy was confirmed by Illumina sequencing.

The CYP3A4*1G, *1B, and *22 alleles were genotyped by direct sequencing. DNA fragments containing the polymorphic site were amplified using PCR, using the forward primer 5′-CTAAACTGTGATGCCCTAC-3′ and the reverse primer 5′-CTTTTCAGAGCCTTCCTA-3′ for *1G; the forward primer 5′-CTCACCTCTGTTCAGGGAAAC-3′ and the reverse primer 5′-ATGGCCAAGTCTGGGATGAG-3′ for *1B; and the forward primer 5′-TTCCTATGATGGGCTCCTTG-3′ and reverse primer 5′-GGAAACCCACATGTCCAGTC-3′ for *22.

### CYP3A4/5 mRNA detection by real-time PCR

Total RNA containing small RNA was isolated from liver tissue in RNAlater^®^ solution using Trizol (Invitrogen Life Technologies, USA) according to the manufacturer’s protocol. The concentrations ofall RNA samples were quantified using NanoDrop 2000 (Thermo Scientific).

Two-step quantitative RT-PCR was performedto detect CYP3A4 and CYP3A5 expression. RNA (1 μg) was reverse transcribed to cDNA using the PrimeScript RT Reagent kit (Takara, Japan) in accordance with the manufacturer’s protocol. In brief, 20 μL reactions that included 4 μl PrimeScript RT Master Mix, nuclease-free water and template RNA were incubated for 15 min at 37 °C and then for 15 s at 85 °C, after which they were stored at −20 °C. The cDNA was then quantified by qPCR on ViiA 7 instrument (Life Technologies, USA) with the appropriate primers (CYP3A4: forward 5′-GGTCAACAGCCTGTGCTGGC-3′ and reverse 5′-CTGGACCAAAAGGCCTCCGGT-3′; CYP3A5: forward 5′-GCTGTCAGCCTGGTGCTCCT-3′ and reverse 5′-TCCAGAGACCCTGACGATAGGACAA-3′) using SYBR Premix Ex TaqII (Takara, Japan). β-actin mRNA was used as the endogenous control with forward primer 5′-CCTGGCACCCAGCACAAT-3′ and reverse primer 5′-GCCGATCCACACGGAGTACT-3′. The following thermal profile was used: initial denaturation at 95 °C for 2 min, followed by 40 cycles of denaturation at 95 °C for 30 s, annealing at 58 °C for 30 s, and extension at 72 °C for 30 s. The melting curve was set from 65 °C to 95 °C, and readings were obtained at 0.5 °C increments for 5 s. All qPCR experiments were run with three replicates. The relative expression level of each mRNA was calculated by the 2^−ΔCt^ method using the equation ΔCt = mean Ct_mRNA_ − mean Ct_β-actin_. Replicates/wells with Ct > 35 were excluded.

### Tissue miRNA selection method and detection by real-time PCR

To reveal the contribution of miRNAs to the variability of atorvastatin metabolism, with focus on accounting for the missing heritability of CYP3A4 and CYP3A5 genes, candidate miRNAs were selected based on the following criteria: (1) all miRNAs were previous experimentally verified to target enzyme genes involved in atorvastatin metabolism (e.g., CYP3A4, CYP3A5, CYP2C8, UGT1A1 and UGT1A3), factors regulating CYP3A4 function (e.g., nuclear receptors), and factors involving inflammatory signaling^32^, and (2) miRNAs were predicted to target CYP3A4 or CYP3A5 mRNA using three computational programs, TargetScan7.0 (http://www.targetscan.org/), miRanda 2010 with all mirSVR (http://www.microrna.org/), and FINDTAR3 (http://bio.sz.tsinghua.edu.cn/) with default thresholds. The predictive binding sites for miRNAs in 3′-UTR fragments of CYP3A4 or CYP3A5 were shown in Supplementary Fig. S8.

Specifically, miR-27b, miR103, miR107 were previously reported to play key roles in regulating CYP3A and CYP2C^20^,^33^, while miR-142 and miR-491 were reported to play key roles in regulating phase II enzymes, namely UGT1A1 and UGT1A3^34^,^35^. PPARα was reported to regulate the expression of CYP3A4^36^ and was found to be regulated by miR-21, miR-27 and miR-130a^37^,^38^. PXR is a well-known nuclear receptor regulating CYP3A4 and was reported to be regulated by miR-27a, miR-27b and miR-371b^39^,^40^. MiR-27b was reported to regulate the transcription of VDR, which regulates CYP3A4 gene expression^20^. LXRα is involved in the basal transcriptional regulation of CYP3A4 in primary human hepatocytes and miR-206 can serve as a negative CYP3A4 regulator by interfering with PXR-activation of CYP3A4 expression^41^,^42^. MiRNAs involved in inflammatory signaling may play a role in regulation of CYP3A expression^32^. miR-106a could regulate IL-10 and IL-8 expression^43^,^44^, while miR-126 is involved in regulation of NF-κB signaling pathway^45^,^46^. MiR-1260b was selected as negative control because it was not expected to bind to CYP3A4 or CYP3A5 mRNA. Therefore, a total of 13miRNAs, namely miR-21-5p, miR-27a-3p, miR-27b-3p, miR-103a-3p, miR-106a-5p, miR-107, miR-126-5p, miR-130a-3p, miR-142-5p, miR-206, miR-371b-5p, miR-491-3p, and miR-1260b, were selected.

The expression levels of 13 miRNAs and reference snRNA U6 were determined by a stem-loop qRT-PCR method. Total RNA (1 μg) was reverse transcribed using the PrimeScript RT Reagent Kit (Takara, Japan) in accordance with the manufacturer’s protocol. In brief, 10 μL reactions included 2 μl PrimeScript RT Buffer, 0.5 μl RT enzyme, 1 μl primer, nuclease-free water and template RNA were incubated for 60 min at 42 °C, and then for 10 min at 79 °C, and stored at −20 °C. qRT-PCR results were analyzed using Bulge-Loop™ miRNA primers (Ribobio Co., Guangzhou, China) and iTaq Universal SYBR Green Supermix (Bio-Rad, USA) in accordance with the manufacturer’s protocol ona Bio-Rad CFX Manager instrument (Bio-Rad). The following thermal profile was used: initial denaturation at 95 °C for 5 min, followed by 40 cycles of denaturation at 95 °C for 10 s and elongation at 55.7 °C for 30 s. A melting curve was set from 65 °C to 95 °C and readings were obtained at 0.5 °C increments for 5 s. The relative expression level of each miRNA was calculated by the 2^−ΔCt^ method using the equation ΔCt = mean Ct_miRNA_ − mean Ct_U6_. Replicates/wells with Ct > 35 were excluded.

### Cell culture

The human embryonic kidney cell line HEK293 and the human hepatocellular carcinoma cell line HepG2 were obtained from Medical Research Center of Guangdong General Hospital. The cryopreserved primary human hepatocytes was obtained from BioreclamationIVT. All cells were cultured in Dulbecco’s modified Eagles medium (DMEM) (Hiclone, USA) with 10% fetal bovine serum (FBS) (Hiclone, USA) and maintained at 37 °C in 5% carbon dioxide.

### Transfection of miRNA mimic and inhibitor into cells

MiR-27b mimic, miR-142 mimic, miR-206 mimic, miR-21 mimic, miR-130a mimic, mimic negative control, miR-27b inhibitor, miR-142 inhibitor, miR-206 inhibitor, miR-21 inhibitor, miR-130a inhibitor, and inhibitor negative control were obtained from RiboBio (Guangzhou, China). MiRNA transfections were performed using Lipofectamine 3000 (Invitrogen Life Technologies, USA) according to the manufacturer’s instruction.

### Analysis of CYP3A4 mRNA in HepG2 cells

Total RNAs from treated HepG2 cellswere prepared after 48 h transfection. Relative expression of CYP3A4 mRNA were performed by real-time RT–PCR.

### CYP3A enzyme activity in cryopreserved primary human hepatocytes

Cryopreserved human hepatocytes were thawed and plated onto collagen-coated 96 wells at a cell density of 0.7 × 10^5^ cells/ml in 0.1 mL/well. After 12 hours, the culture medium was changed with serum-free medium and transfection of miRNA mimics and inhibitors was conducted. After the 48 h treatment period, CYP3A4 enzyme activity in cryopreserved human hepatocyteswas determined using the P450-Glo™ CYP3A4 assay (Promega, USA), which contained the substrate luciferin isopropyl acetal (luciferin-IPA) and luciferin detection reagent, following the manufacturer’s protocol.

### Reporter Plasmid Construction

Eight luciferase reporter plasmids were constructed. Human CYP3A4 3′-UTR fragments, the sequence from 1620 to 2792 (~1173 bp) in the human CYP3A4 mRNA (NM_017460.5), containing a putative miR-27b/miR-206/miR-21/miR-130a binding sites or mutated binding sites (reverse binding sites), and human CYP3A5 3′-UTR fragments, the sequence from 552 to 1105(~554 bp) in the human CYP3A5 mRNA (NM_001190484.2), containing a putative miR-27b/miR-142binding sites or mutated binding sites (reverse binding sites), were cloned into the pmiR-RB-REPORT^TM^ Vector (Ribobio Co., Guangzhou, China) downstream of the renilla luciferase reporter gene. The pmiR-RB-REPORT^TM^ Vector also includes an independent firefly luciferase reporter gene, which functions as a control for transfection efficiency. Multiple binding sites were mutated in has-miR-130a because it has three predicted targets. All constructs were verified by sequencing.

### Luciferase Reporter Gene Assay

The luciferase reporter plasmids were co-transfected with the corresponding miRNA mimic into HEK293 cells. Briefly, the day before transfection, the HEK293 cells were seeded into 96-wellplates with 2 × 10^4^ cells/well and cultured at 37 °C in a humidified incubator with 5% CO_2_ for 24 h. Cells were co-transfected with 100 ng of reporter plasmid and 50 nM of the corresponding miRNA mimic or miRNA mimic negative control using lipofectamine^®^ 3000 (Invitrogen Life Technologies, USA). After incubationfor 48 h, the luciferase activity was measured with a luminometer (Infinite^®^ F500 Multimodel Plate Reader, TECAN) using the Dual-Glo^®^ Luciferase Assay System (Promega, USA) with the manufacturer’s protocol. The results were expressed as the relative ratio of *Renilla* activity/firefly activity. Each experiment was performed with at least three replicates. The difference between the miRNA group and the control was evaluated using a t-test.

### Statistical analysis

Demographic and clinical characteristics were summarized using counts (percentages) for categorical variables and mean ± standard deviation for continuous variables. The normality of distribution of continuous variables was checked by the Shapiro-Wilk test. If the ranges of the dependent variables were skewed, logarithmic transformation was performed before analysis. Spearman correlation coefficients were calculated to describe the correlations between miRNAs and CYP3A4 and CYP3A5 mRNA expression and CYP3A enzyme activity in human liver tissues. The Mann-Whitney U test was used to compare differences between two groups, and the Kruskal-Wallis test was used to compare differences among three groups.

To identify the independent contribution of each of the factors to interindividual variability in CYP3A4 and CYP3A5 gene expression, CYP3A activity, and AT metabolism, multivariate linear regression analysis with calculation of the coefficient of determination (R^2^) was performed. Variables with P < 0.10 were entered into the multivariable model and only variables with P < 0.05 were retained in the model. Variables of variant alleles were coded as 0, 1, and 2 for zero, one, and two copies of the variant allele, respectively. Because the minor genotype frequency of CYP3A5*1/*1 was less than 5%, the rare genotype was combined with the intermediate genotype. Variables included age, sex (coded as 0/1), liver cancer (coded as 0/1), and medical history (coded as 0/1). A false discovery rate (FDR) control was used to correct for multiple comparisons of miRNAs using the SAS PROC MULTTEST with the FDR option (SAS Institute lnc., Cary, North Carolina, USA). P values less than 0.05 were considered statistically significant, and FDR was controlled to the 0.05 level.

## Additional Information

**How to cite this article**: Liu, J.-E. *et al*. The independent contribution of miRNAs to the missing heritability in CYP3A4/5 functionality and the metabolism of atorvastatin. *Sci. Rep.* **6**, 26544; doi: 10.1038/srep26544 (2016).

## Supplementary Material

Supplementary Information
