# Supplementary material for: The independent contribution of miRNAs to the missing heritability in CYP3A4/5 functionality and the metabolism of atorvastatin
Source: Sci Rep. 2016 May 23;6:26544. doi: 10.1038/srep26544 (PMC4876377; doi:10.1038/srep26544)
Supplement: Supplementary Information [file srep26544-s1.pdf]

Supplementary Information

The independent contribution of miRNAs to the missing heritability in CYP3A4/5 functionality and the metabolism of atorvastatin

Ju-E Liu[1,2,#], Bin Ren[1,#], Lan Tang[3,#], Qian-Jie Tang[2,4], Xiao-Ying Liu[2], Xin Li[2,5], Xue Bai[2,6], Wan-Ping Zhong[2,3], Jin-Xiu Meng[2], Hao-Ming Lin[7], Hong Wu[7], Ji-Yan Chen[2,8], Shi-Long Zhong[2,8,*]

#Ju-E Liu, Bin Ren and Lan Tang contribute equally to this paper

1. Department of Pharmacy, The First Affiliated Hospital, Sun Yat-sen University, Guangzhou, Guangdong 510080, China
2. Medical Research Center, Guangdong General Hospital, Guangzhou, Guangdong 510080, China
3. Department of Pharmaceutics, School of Pharmaceutical Sciences, Southern Medical University, Guangzhou 510515, China.
4. Institute of Chinese medical science, Guangdong TCM key Laboratory for metabolism，Guangdong pharmaceutical university, Guangzhou 510006, China
5. Department of Pharmacology, School of Pharmaceutical Sciences, Guangzhou Medical University, Guangzhou 511436, China
6. School of pharmaceutical science, Sun Yat-Sen University, Guangzhou, Guangdong 510006, China
7. Department of Hepatobiliary Surgery, Sun Yat-Sen Memorial Hospital, Sun Yat-sen University, Guangzhou 510120, China
8. Guangdong Cardiovascular Institute, Guangdong Academy of Medical Sciences, Guangzhou, Guangdong 510080, China

***Correspondence to**

Shi-Long Zhong, Ph.D.

Professor of Medical Research Center,Guangdong General Hospital,

Guangdong Cardiovascular Institute,

Guangdong Academy of Medical Sciences.

106 Zhongshan Road, Weilun Bldg.1112,

Guangzhou 510080, P. R. China

Tel: +8620-83827812 - 51157

Email: zhongsl@hotmail.com

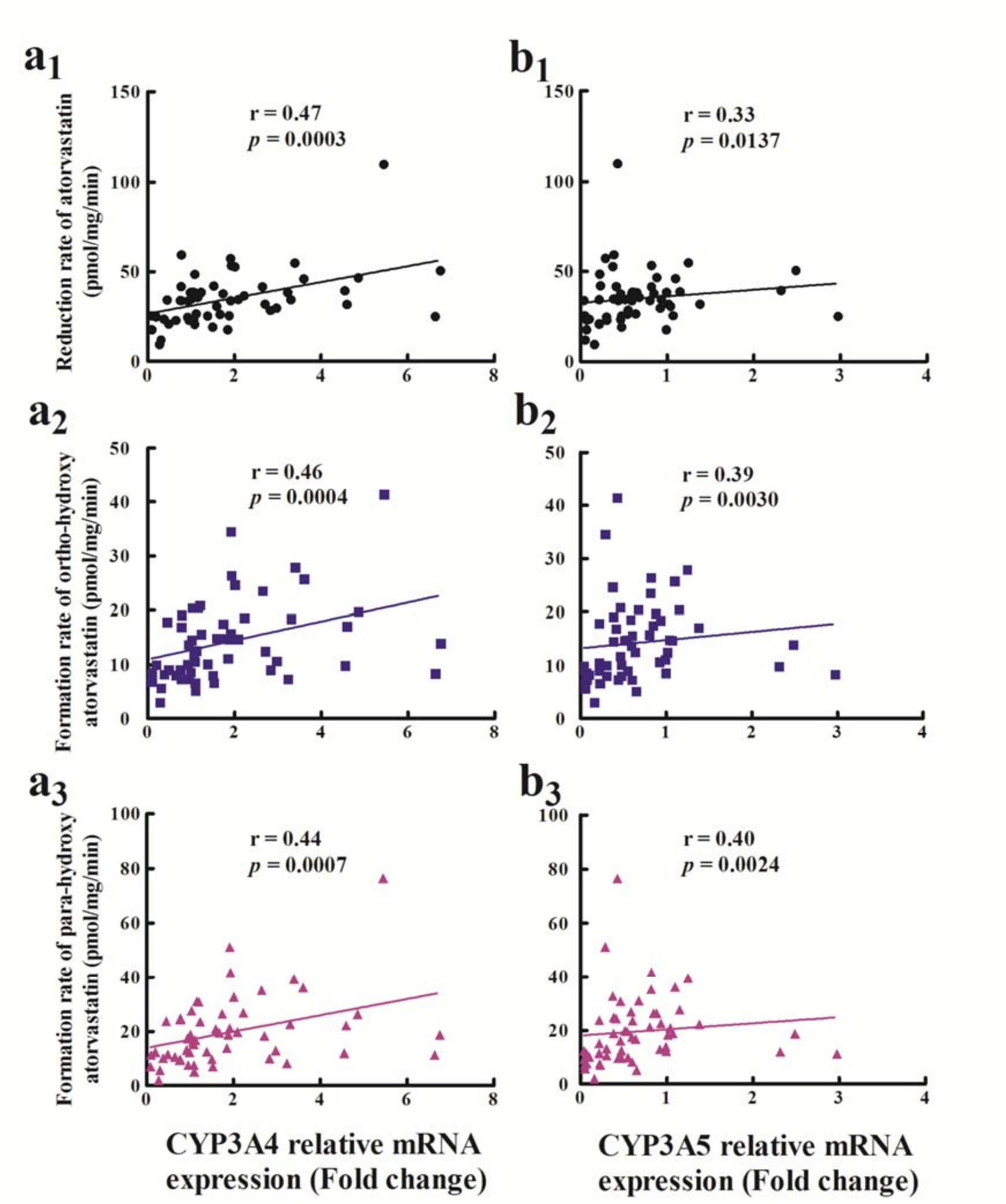

Supplementary Figure S1. Correlation between CYP3A gene expression and the reduction rate of atorvastatin or the formation rate of two metabolites of atorvastatin. The expression of CYP3A4 was significantly associated with reduction rate of atorvastatin (**a_1**), formation rate of ortho-hydroxy atorvastatin (**a_2**), formation rate of para-hydroxy atorvastatin (**a_3**). Expression of CYP3A5 was associated with reduction of atorvastatin (**b_1**), formation of ortho-hydroxy atorvastatin (**b_2**), and formation of para-hydroxy atorvastatin (**b_3**).

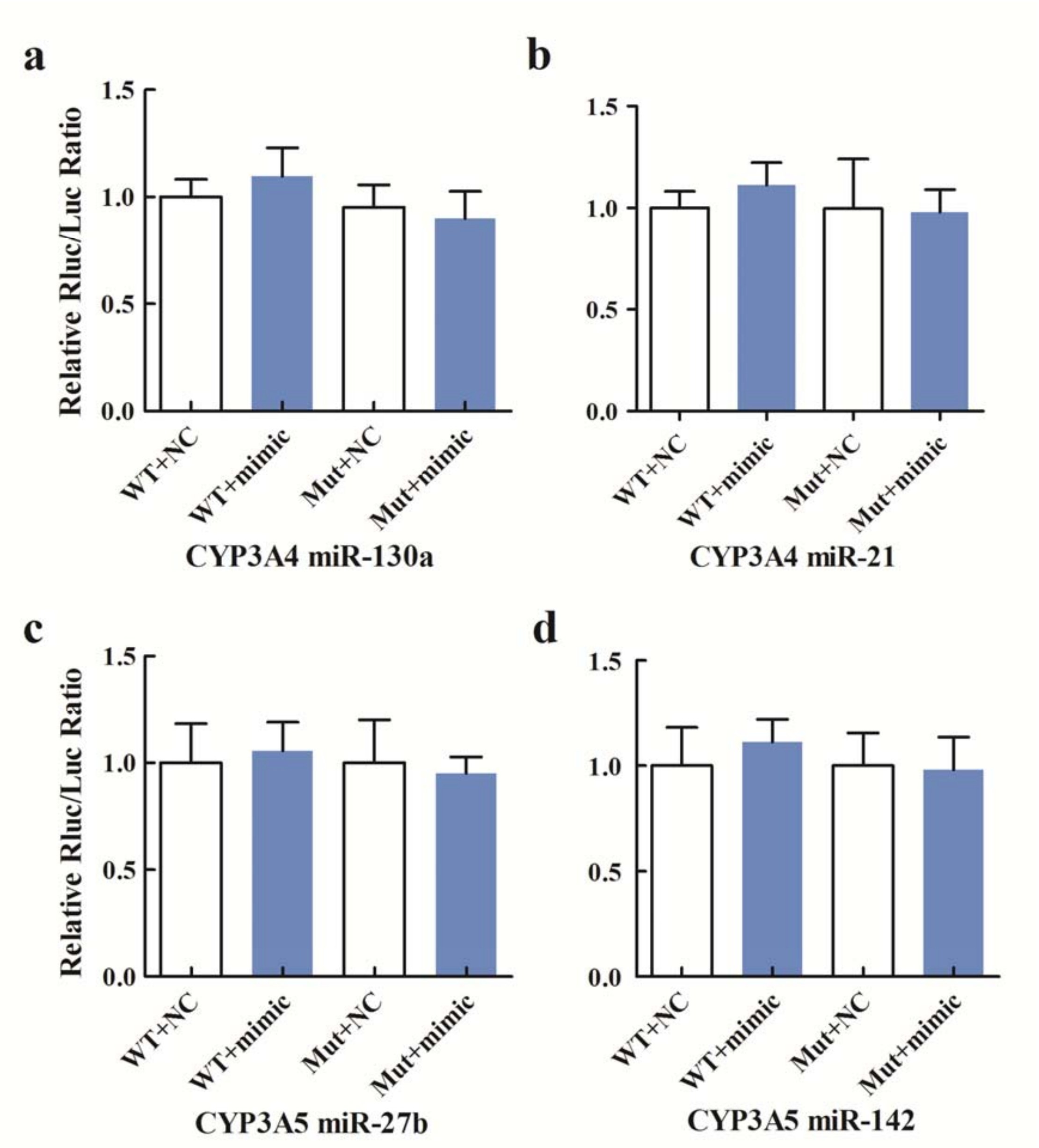

Supplementary Figure S2. Luciferase reporter assay to evaluate the direct inhibition of miR-130a, miR-21, miR-27b and miR-142 on CYP3A4 or CYP3A5 expression. (a) miR-130a did not target the predicted binding sites in the CYP3A4 3'-UTR. (b) miR-21 did not target the predicted binding sites in the CYP3A4 3'-UTR. (c) miR-27b did not target the predicted binding sites in the CYP3A5 3'-UTR. (d) miR-142 did not target the predicted binding sites in the CYP3A5 3'-UTR.

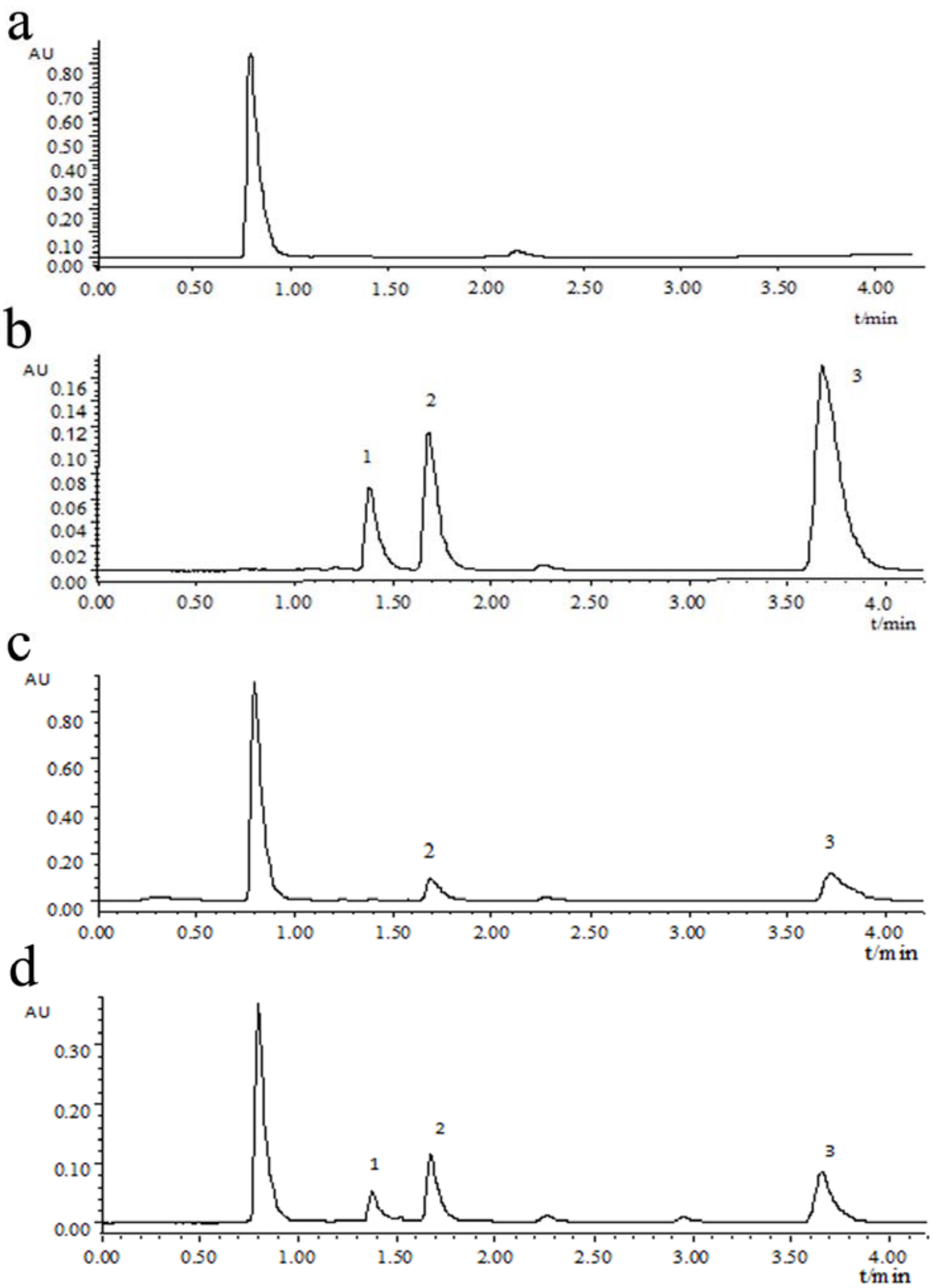

Supplementary Figure S3. The chromatograms of testosterone and its metabolite. (**a**) blank microsomal; (**b**) reference standards; (**c**) inactive microsomal incubation mixtures; (**d**) active microsomal incubation mixtures. 1, 2 and 3 represent 6β-hydroxytestosterone, internal standard hydrocortisone and testosterone, respectively.

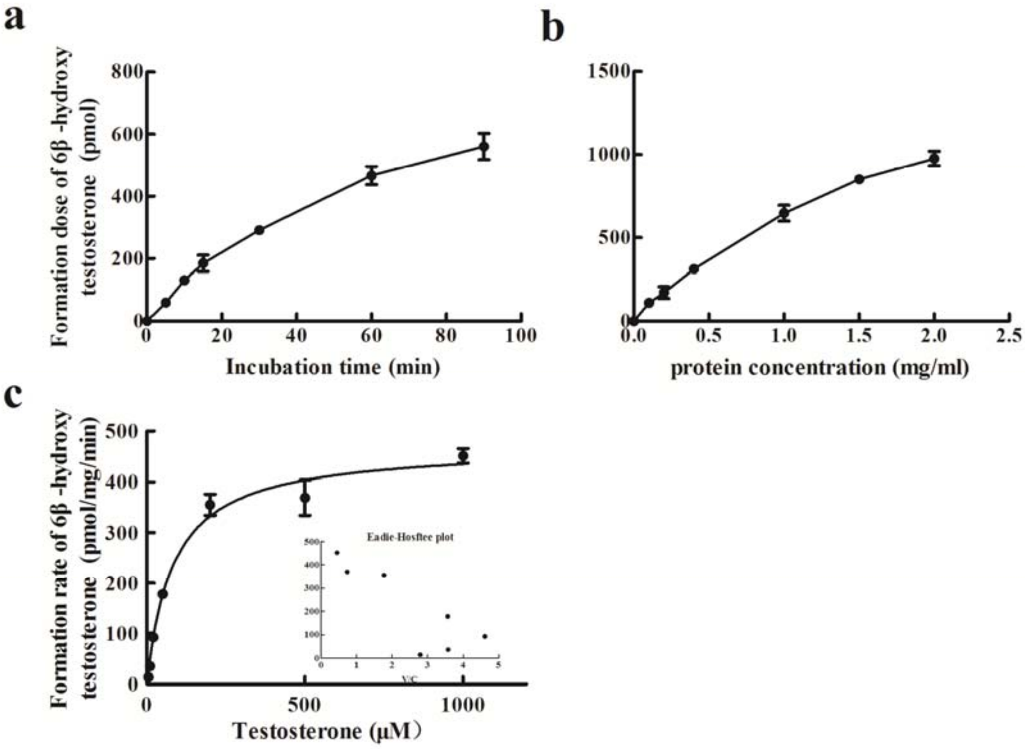

Supplementary Figure S4. Effect of testosterone incubation time and microsomal protein concentration on the formation of 6β-OHT by human liver microsomes and enzyme kinetic parameters. (**a**) Time-dependent formation of 6β-OHT in human liver microsome. (**b**) Microsomal protein concentration-dependent formation of 6β-OHT. (**c**) Kinetic profiles of 6β-OHT activity in HLM with obtained K_m= 83.06 ± 14.93μM, V_{max}= 470.7 ± 22.0 pmol/mg/min. Human liver microsomes from a pooled sample were incubated with testosterone (20 μM) for different times (0-90 min with 0.5 mg/ml protein) or at different protein concentrations (0-2.0mg/ml for 20 min). Data shown are the average mean ± S.D. of three independent experiments.

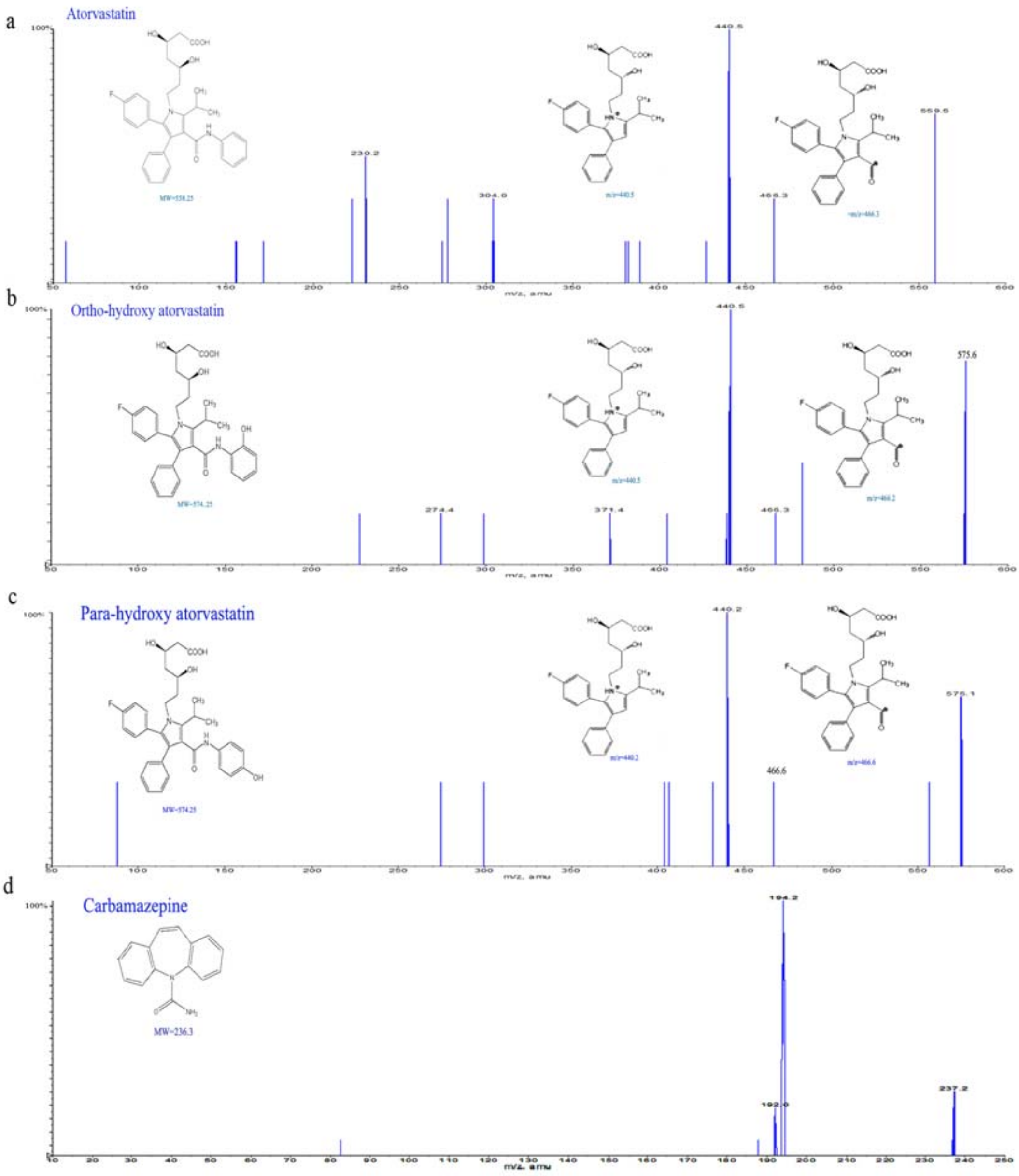

Supplementary Figure S5. Product ion spectra of atorvastatin (A), ortho-hydroxy atorvastatin (B), para-hydroxy atorvastatin (C), and carbamazepine (D).

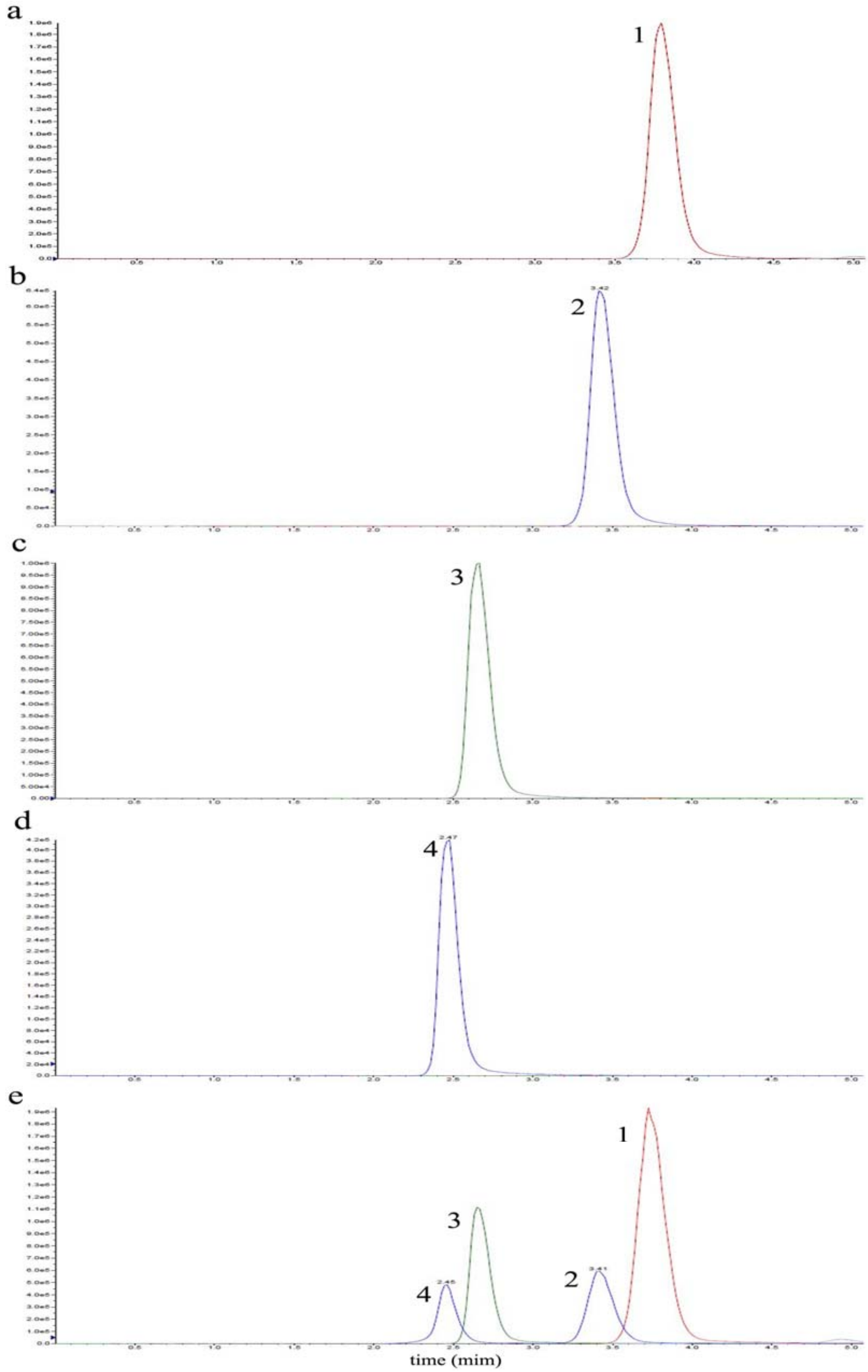

Supplementary Figure S6. The chromatograms of atorvastatin and its metabolite. (**a**) atorvastatin; (**b**) ortho-hydroxy atorvastatin; (**c**) para-hydroxy atorvastatin; (**d**) internal standards carbamazepine; (**e**) mixtures. 1, atorvastatin; 2, ortho-hydroxy atorvastatin; 3, internal standard; 4, para-hydroxy atorvastatin.

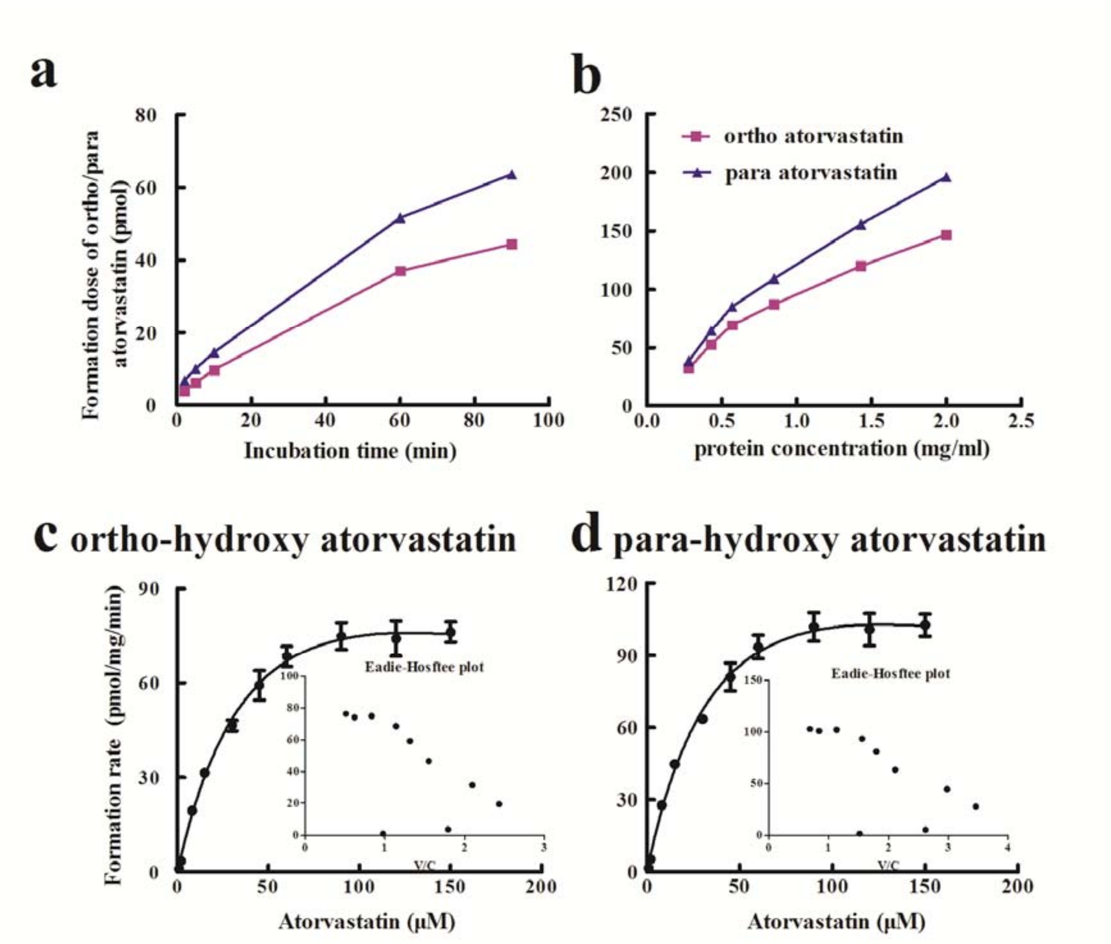

Supplementary Figure S7. Enzyme kinetics and effect of atorvastatin incubation time and microsomal protein concentration on atorvastatin metabolism by human liver microsomes. (**a**) Time-dependent formation of ortho- and para-hydroxy atorvastatin in human liver microsomes. (**b**) Microsomal protein concentration-dependent formation of ortho- and para-hydroxy atorvastatin. Enzyme kinetics of the formation rates of (**c**) ortho- and (**d**) para-hydroxy atorvastatin from atorvastatin in pooled human liver microsomes. Human liver microsomes from a pooled sample were incubated with testosterone (1-150 μM) with 0.3 mg/ml protein for 60 min at 37 ℃. Data shown are the average mean ± S.D. of triplicate incubations.

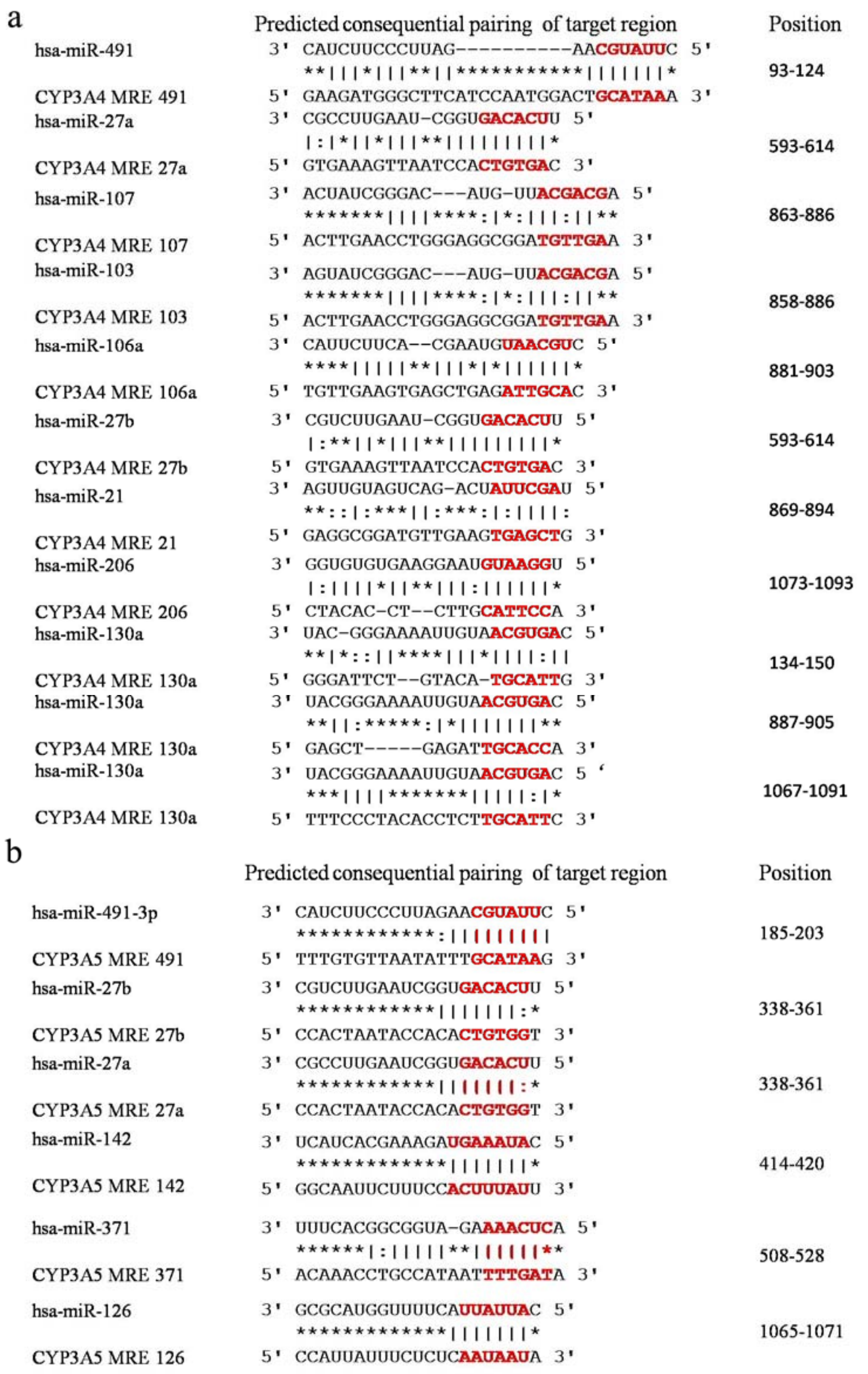

Supplementary Figure S8. MiRNA recognition element within CYP3A4 or CYP3A5 3'-UTR predictive by TargetScan, FINDTAR3 and miRanda. (a) predictive binding sites of miRNAs within CYP3A4 3'-UTR. (b) predictive binding sites of miRNAs within CYP3A5 3'-UTR

Supplementary Table 1. Baseline clinical characteristics and CYP3A4/5 genotypes impact on gene expression, CYP3A activity and metabolism of atorvastatin

	**Aatorvastatin**		**Ortho-hydroxy atorvastatin**		**Para-hydroxy atorvastatin**		**CYP3A4 mRNA**		**CYP3A5 mRNA**		**CYP3A activity**	
**Factors**	**B or Mean±SD**	***P***	**B or Mean±SD**	***P***	**B or Mean±SD**	***P***	**B or Mean±SD**	***P***	**B or Mean±SD**	***P***	**B or Mean±SD**	***P***
Sex(Male)	-10.49	0.017	-2.95	0.183	-5.45	0.149	-0.281	0.544	0.042	0.810	-86.73	0.137
Age (year)	-0.13	0.437	-0.06	0.438	-0.14	0.290	-0.028	0.085	-0.007	0.292	-2.05	0.335
Concomitant enzyme inducer	7.82	0.184	1.98	0.501	3.77	0.465	0.002	0.998	-0.283	0.208	29.75	0.703
Liver cancer	-3.38	0.416	-0.74	0.722	-1.76	0.619	-0.775	0.066	-0.285	0.070	-37.45	0.492
Hepatitis B	-2.69	0.600	-0.51	0.841	-1.26	0.773	-0.402	0.454	-0.038	0.853	-69.51	0.313
CYP3A5												
*1/*1 or *1/*3	38.41±9.55	0.012	16.19±6.77	0.038	22.87±10.44	0.022	2.412±1.849	0.044	0.980±0.719	0.001	285.9±124.9	0.029
*3/*3	32.52±17.78		12.94±7.86		17.49±14.04		1.536±1.573		0.456±0.351		243.6±236.6	
CYP3A4												
*1/*1	34.19±19.43		13.81±8.76		18.86±15.45		1.530±1.217		0.484±0.353		266.2±249.6	
*1/*1G	36.00±9.85	0.500	15.37±6.17	0.194	21.35±9.81	0.234	2.118±1.700	0.222	0.764±0.619	0.033	258.5±130.4	0.654
*1G/*1G	33.14±6.17		10.82±5.32		15.34±9.19		3.046±2.484		1.372±1.079		231.8±165.9	

Supplementary Table 2. Impact of miRNAs on CYP3A gene expression and activity, and atovastatin metabolism.

microRNA	CYP3A4 mRNA			CYP3A5 mRNA			CYP3A activity			Aatorvastatin			Ortho-hydroxy atorvastatin			Para-hydroxy atorvastatin		
	**r**	***P***	***FDR***	**r**	***P***	***FDR***	**r**	***P***	***FDR***	**r**	***P***	***FDR***	**r**	***P***	***FDR***	**r**	***P***	***FDR***
miR-27b	-0.37	0.006	0.020	-0.25	0.068	0.221	-0.50	0.000	0.007	-0.38	0.004	0.022	-0.43	0.001	0.013	-0.46	0.001	0.013
miR-206	-0.28	0.040	0.104	-0.21	0.129	0.335	-0.38	0.005	0.016	-0.42	0.001	0.013	-0.36	0.007	0.030	-0.39	0.003	0.013
miR-103	-0.02	0.908	0.910	0.04	0.791	0.861	0.00	0.990	0.990	-0.11	0.431	0.560	0.02	0.870	0.943	0.02	0.893	0.910
miR-107	-0.02	0.910	0.910	0.03	0.839	0.861	-0.01	0.951	0.990	-0.06	0.655	0.710	-0.01	0.949	0.949	-0.02	0.910	0.910
miR-371	-0.13	0.362	0.471	-0.06	0.664	0.861	-0.09	0.535	0.696	-0.07	0.624	0.710	-0.05	0.697	0.824	-0.09	0.515	0.670
miR-491	-0.11	0.417	0.493	-0.04	0.800	0.861	0.04	0.778	0.920	-0.03	0.819	0.819	0.07	0.604	0.785	0.03	0.813	0.910
miR-1260	-0.24	0.082	0.152	-0.14	0.297	0.499	-0.23	0.087	0.126	-0.13	0.362	0.523	-0.16	0.259	0.374	-0.19	0.170	0.246
miR-21	-0.46	0.000	0.003	-0.31	0.022	0.095	-0.35	0.009	0.023	-0.37	0.005	0.022	-0.32	0.018	0.059	-0.35	0.010	0.033
miR-27a	-0.27	0.049	0.106	-0.18	0.180	0.390	-0.38	0.005	0.016	-0.27	0.045	0.117	-0.31	0.024	0.062	-0.33	0.014	0.036
miR-106	-0.16	0.253	0.365	-0.02	0.861	0.861	-0.28	0.040	0.074	-0.23	0.094	0.204	-0.27	0.050	0.108	-0.28	0.040	0.087
miR-126	-0.22	0.114	0.185	-0.14	0.307	0.499	-0.24	0.073	0.119	-0.15	0.285	0.463	-0.20	0.149	0.242	-0.24	0.078	0.127
miR-130a	-0.45	0.001	0.004	-0.32	0.016	0.095	-0.40	0.002	0.013	-0.31	0.020	0.065	-0.36	0.007	0.030	-0.39	0.003	0.013
miR-142	-0.49	0.000	0.003	-0.32	0.005	0.065	-0.31	0.020	0.043	-0.22	0.116	0.215	-0.24	0.085	0.158	-0.25	0.066	0.123

Supplementary Table 3. Multiple regression analysis with forward selection for modeling metabolism of atorvastatin.

Dependent variable	Parameter	Estimate	Partial R^2	Model R^2	P value
CYP3A4 mRNA					
	Intercept	2.459			
	miRNA142	-231.952	0.122	0.122	0.002
	liver cancer	-0.999	0.102	0.224	0.010
	CYP3A4*1G	0.649	0.069	0.293	0.032
CYP3A5 mRNA					
	Intercept	1.245			
	CYP3A5*3	-0.474	0.189	0.189	0.001
	miRNA142	-75.145	0.094	0.282	0.005
	liver cancer	-0.304	0.067	0.349	0.028
CYP3A activity					
	Intercept	5.401			
	miR-27b	-29.334	0.200	0.200	0.002
	CYP3A4 mRNA	0.158	0.095	0.295	0.009
	miR-206	131325.000	0.058	0.353	0.037
Atorvastatin					
	Intercept	19.621			
	CYP3A activity	0.059	0.600	0.600	<.0001
Ortho-hydroxy atorvastatin					
	Intercept	5.538			
	CYP4A activity	0.034	0.788	0.788	<.0001
Para-hydroxy atorvastatin					
	Intercept	4.288			
	CYP5A activity	0.059	0.839	0.839	<.0001
